# Structural Similarity with Cholesterol Reveals Crucial Insights into Mechanisms Sustaining the Immunomodulatory Activity of the Mycotoxin Alternariol

**DOI:** 10.3390/cells9040847

**Published:** 2020-03-31

**Authors:** Giorgia Del Favero, Raphaela M. Mayer, Luca Dellafiora, Lukas Janker, Laura Niederstaetter, Chiara Dall’Asta, Christopher Gerner, Doris Marko

**Affiliations:** 1 Department of Food Chemistry and Toxicology, Faculty of Chemistry, University of Vienna Währinger Straße 38-40, 1090 Vienna, Austria; mayer.m.raphaela@gmail.com (R.M.M.); doris.marko@univie.ac.at (D.M.); 2 Core Facility Multimodal Imaging Faculty of Chemistry, University of Vienna Währinger Straße 38-40, 1090 Vienna, Austria; 3 Department of Food and Drugs, University of Parma, 43121 Parma, Italy; luca.dellafiora@unipr.it (L.D.); chiara.dallasta@unipr.it (C.D.); 4 Department of Analytical Chemistry, Faculty of Chemistry, University of Vienna, Währinger Straße 38, 1090 Vienna, Austria; lukas.janker@univie.ac.at (L.J.); laura.niederstaetter@univie.ac.at (L.N.); 5 Joint Metabolome Facility, Faculty of Chemistry, University of Vienna. Währinger Straße 38-40, 1090 Vienna, Austria

**Keywords:** immunomodulation, alternariol, membrane, cholesterol

## Abstract

The proliferation of molds in domestic environments can lead to uncontrolled continuous exposure to mycotoxins. Even if not immediately symptomatic, this may result in chronic effects, such as, for instance, immunosuppression or allergenic promotion. Alternariol (AOH) is one of the most abundant mycotoxins produced by *Alternaria alternata* fungi, proliferating among others in fridges, as well as in humid walls. AOH was previously reported to have immunomodulatory potential. However, molecular mechanisms sustaining this effect remained elusive. In differentiated THP-1 macrophages, AOH hardly altered the secretion of pro-inflammatory mediators when co-incubated with lipopolysaccharide (LPS), opening up the possibility that the immunosuppressive potential of the toxin could be related to an alteration of a downstream pro-inflammatory signaling cascade. Intriguingly, the mycotoxin affected the membrane fluidity in macrophages and it synergistically reacted with the cholesterol binding agent MβCD. In silico modelling revealed the potential of the mycotoxin to intercalate in cholesterol-rich membrane domains, like caveolae, and immunofluorescence showed the modified interplay of caveolin-1 with Toll-like Receptor (TLR) 4. In conclusion, we identified the structural similarity with cholesterol as one of the key determinants of the immunomodulatory potential of AOH.

## 1. Introduction

**Figure 1:**
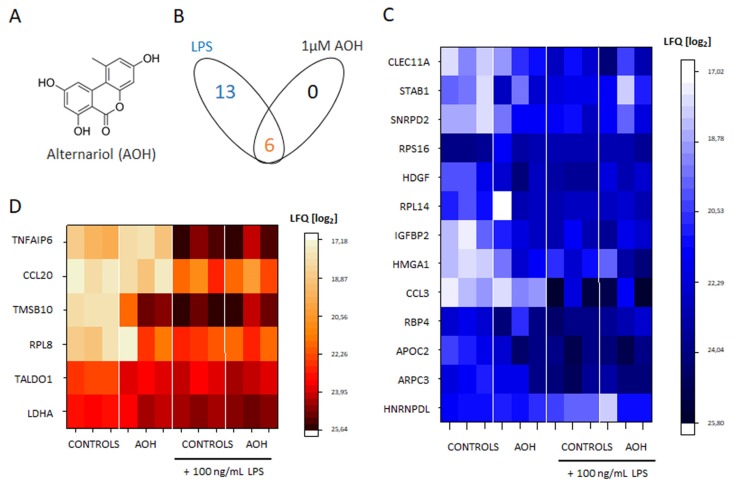
(**A**) Structure of alternariol AOH. (**B**) Significantly regulated proteins in the secretome of THP-1 macrophages, after 5 h incubation with 100 ng/mL LPS, 1 μM AOH or combination of the two. (**C**) Thirteen proteins regulated exclusively by incubation with 100 ng/mL LPS. (**D**) Six proteins regulated by co-incubation with 100 ng/mL LPS and 1 μM AOH. The heat map depicts the three biological replicates performed for each treatment group.

Awareness regarding the risk associated with exposure to environmental contaminants is often very limited, especially when exposure occurs inconspicuously in a domestic environment and the health consequences are spread over a broad timeframe. Increasing proof of evidence indicates a correlation between residential mold exposures with health effects on the respiratory tract [1], and mycotoxins are suspected to play a major role in immune-related diseases like asthma [2]. Other studies underpin the immunomodulatory potential of several fungal compounds [3,4,5,6,7,8]. Hence, there is an urgent need to better understand the molecular mechanisms mediating these effects for proper toxicological evaluation and eventually, therapeutic treatment. *Alternaria alternata* is a typical species identified in domestic environments [9] and it is known to produce hundreds of different secondary metabolites, many of them already classified as mycotoxins [10]. Among the most studied secondary metabolites produced by *Alternaria alternata* is the dibenzopyrone alternariol (AOH, Figure 1A). In our study, we decided to focus on relatively low concentrations (0.1–1 μM), with the idea of trying to isolate potential effects of non-toxic dose-exposure. In commercial products, AOH can be found in concentrations up to 20 ng/g [11]. When administered orally to rats, AOH can be detected in urine and feces [10], suggesting the potential for systemic bioavailability. The immunomodulatory potential of the compound has already been described, reporting the pro-inflammatory effects on skin cells [12] and induction of senescence and cell cycle arrest in RAW264.7 mouse macrophages [5,13,14]. Immunosuppressive potential was also related to the capability to suppress LPS-induced activation of macrophages [3,6,7] as well as to modify cytokines transcription and release in intestinal Caco-2 cells [15]. However, key molecular mechanisms determining these responses are still under debate. Receptor-activated signaling (i.e., Toll-like receptor 4, TLR4) plays a central role in sustaining LPS-induced inflammatory cascades and such activity is highly dependent on receptor functionality, as well as on a sophisticated structural organization within the cell membrane [16]. Membrane composition (i.e., rafts and caveolae) can be crucial for receptor distribution and turnover [17]. In addition, mechanisms regulating immune responses are subject to intensive research, since acute and chronic pathologies involving the regulation of the immune system are continuously increasing [18]. In line with this, immunomodulatory action can derive, for instance, from the interaction with receptors [19], or from the modulation of the structure/biophysical properties of the surrounding environment [20]. It was recently demonstrated that membrane oxidation can interfere with pro-inflammatory signals triggered by LPS [21]. Lipid composition and the oxidation status can tune the down-streaming cascade of LPS, inducing pro-inflammatory responses [22]. Similarly, toxicants can exert their effect through direct interaction with the pro/anti-inflammatory cascade or indirectly via the modulation of cell biophysical properties. In line with this, the characterization of molecular mechanisms is central, in order to suggest proper therapeutic approaches and supportive interventions.

Taking this as a starting point, we investigated the possibility that the immunomodulatory action of AOH could be already initiated at the membrane level. To this aim, we used the THP-1 human monocytic leukemia cell line, which can be differentiated into macrophages [6,23]. The THP-1 cells have been extensively used to study monocytes/macrophages’ functions and signaling pathways, and are an established model for the study of the immunomodulatory cascades in vitro [24]. We combined secretome analysis (proteomic and eicosanoid profiling) with live cell imaging/image analysis workflows and in silico modelling in order to study the effects of the toxin at membrane level. Indeed, thanks to this approach, we were able to relate AOH structural similarity with cholesterol as a key determinant of its immunomodulatory potential.

## 2. Materials and Methods

### 2.1. Cell Culture Conditions and Reagents

THP-1 monocytes were purchased from (ATCC^®^ TIB202™) and kept in culture in RPMI 1640 Medium supplemented 10% *v*/*v* heat-inactivated fetal calf serum (FCS) and 1% *v*/*v* penicillin/streptomycin (P/S). Macrophage differentiation was induced through incubation with phorbol-12-myristate-13-acetate (PMA, 10 ng/mL) for 72 h, followed by an additional 24 h in PMA free medium, as previously described [6,23]. LPS stimulation 100 ng/mL and solvent controls (controls) DMSO 0.1%.

### 2.2. Supernatant Analysis of Eicosanoids and Proteomics

The cells were seeded in a T25 cell culture flask at 80% confluence with the complete medium. After 3 h incubation the supernatant was removed, spiked with 10–100 nM of each internal standard, centrifuged with 726× *g* and 3 mL precipitated with 12 mL ethanol (EtOH, abs. 99%; AustroAlco), and stored at −20 °C over night. Precipitated proteins were eliminated, and the eicosanoids were further processed from the supernatants. The cells were washed twice with Phosphate Buffer Saline (PBS) and cells were incubated with serum-free medium, containing the stimuli of interest. After additional 2 h incubation the supernatant was removed, centrifuged, and precipitated in ethanol overnight to obtain the secreted proteins.

### 2.3. Protein Sample Preparation

The proteins were used for a filter-assisted protein digest, as described previously [25]. Briefly, the isolated proteins were centrifuged at 4536×*g* for 30 min and the protein pellet dried. After dissolving in sample buffer, the protein concentration was determined with a Bradford assay and 20 µg of total protein was used for the digestion. After reduction with dithiothreitol and alkylation with iodacetamid (both Sigma-Aldrich, Vienna, Austria), proteins were digested with Trypsin/Lys-C (MS grade; Promega Corporation, Madison, WI, USA) and dried via vacuum centrifugation.

### 2.4. Lipids Sample Preparation

Samples were centrifuged (30 min, 4536×*g*, 4 °C) and the supernatant was transferred to new 15 mL Falcon tubes. Ethanol was eliminated via SpeedVac (37 °C), until the original sample volume was restored. Samples were loaded on conditioned 30 mg/mL StrataX solid phase extraction (SPE) columns (Phenomenex, Torrance, CA, U.S.A.) Columns were washed with 2 mL MS grade water and eicosanoids were eluted with 500 µL methanol (MeOH abs.; VWR International, Vienna, Austria) containing 2% formic acid (FA; Sigma-Aldrich). MeOH was evaporated using N_2_ stream at room temperature and reconstituted in 150 µL reconstitution buffer (H_2_O/ACN/MeOH + 0.2% FA - 65:31,5:3,5), containing a set of internal eicosanoid standards at a concentration of 10–100 nM.

### 2.5. Membrane Fluidity

Membrane fluidity was measured as previously described [23], with slight modifications. Differentiated THP-1 cells were incubated for 1 h at 37 °C with 10 μM 1-pyrenedecanoic acid (PDA), diluted in live cell imaging solution. After challenge with the stimuli of interest, fluorescence was measured with a Cytation™ 3 Cell Imaging Multi-Mode Reader equipped with the Gen5™ Data Analysis Software (BioTek Instruments, Inc., Winooski, Vermont, USA). The signal was expressed as a ratio between ex/em. 344/470 nm for the PDA excimers, and ex/em. 344/375 for the monomeric form. Data are the mean of eight independent experiments, performed in quadruplicates.

### 2.6. Live Cell Imaging: Membrane Morphology

For membrane visualization, THP-1 macrophages were incubated with with CellMask™ Deep Red Plasma Membrane Stain for 15 min (1:1000 dilution, depicted in white). Staining solution was diluted in Live Cell Imaging Solution (all reagents were purchased by Molecular Probes, Life Technologies, Thermo Fisher Scientific, Waltham, MA, USA). At the end of the staining, cells were rinsed and maintained in Live Cell Imaging Solution for the microscopy analysis.

### 2.7. Live Cell Imaging: Mitochondrial Superoxide

For superoxide quantification, cells were incubated for 15 min with MitoTracker^®^ Green FM (1:1000 dilution, depicted in blue, indicated as MitoTracker), and MitoSOX™ Red mitochondrial superoxide indicator (1:1000 dilution, red to white, indicated as MitoSOX), as previously described [24]. All reagents were purchased by Molecular Probes, Life Technologies, Thermo Fisher Scientific, Waltham, MA, USA. For the evaluation of the signal intensity, regions of interest (ROI) were selected using the MitoTracker signal as reference, and data were expressed as MitoSox/MitoTracker ratio. Staining solutions were diluted in Live Cell Imaging Solution. At the end of the staining, cells were rinsed and maintained in Live Cell Imaging Solution for the microscopy analysis.

### 2.8. Immunofluorescence

For immunofluorescence experiments [23,26], cells were processed as previously described, with slight modifications [23,26]. Briefly, THP-1 macrophages were incubated for 1 h and immediately fixed with pre-warmed 3.7% formaldehyde (15 min, room temperature). After permeabilization (0.2% Triton-X-100, 10 min), unspecific binding sites were blocked (2% Donkey Serum, 0.5% Bovine Serum Albumine, 1 h). Afterwards, cells were incubated for 2 h with anti-Caveolin-1 antibody (Abcam, ab192452, 1:500), anti-TLR4 antibody [76B357.1] (Abcam, ab22048, 1:200) and anti MIF antibody (Abcam, ab7207, 1:500). After 5 washing steps, species-specific fluorescence-labeled antibodies were used for localization (Donkey Anti-Goat IgG (H+L), Alexa Fluor^®^ 647 (705-605-003) and Donkey Anti-Mouse IgG (H+L) Alexa Fluor^®^ 488 (715-545-150) from Jackson ImmunoResearch Laboratories, Inc., Pennsylvania, US and Donkey anti-Rabbit IgG (H+L) polyclonal secondary antibody, Alexa Fluor^®^ 568 (A10042) from Thermo Fisher Scientific, Waltham, US).

### 2.9. Microscopy

Images were acquired with a Zeiss LSM 710 Confocal Microscope with a ELYRA PS.1 system, for super-resolution with a Plan-Apochromat 63x/1.2 water objective for live cell imaging and a Plan Apochromat 63X/1.4 oil objective for the immunofluorescence experiments. Images were analyzed with the software ZEN Zeiss. If not otherwise specified, optical fields/region of interest (ROI) were quantified for every experimental setup, from at least 3–4 independent experiments.

### 2.10. Statistical Analysis

Data were evaluated with the software OriginPro 2018b (OriginLab Corporation, Northampton, MA, USA). Multiple comparisons of independent samples were performed with a one-way ANOVA test followed by a Fisher test. Student’s *t*-tests were applied for the direct comparison of groups of data. Distributions were considered different, using threshold values of 0.05.

### 2.11. Data Analysis Secretome

For the HPLC-MS/MS analysis, the peptides were resolved in 5 µL 30% formic acid and diluted with 40 µL of mobile phase A (97.9% H_2_O, 2% acetonitrile, 0.1% formic acid). Then, 1 µL for the supernatant samples and 10 µL of cytoplasmic and nuclear samples were injected into the Dionex UltiMate 3000 RSLCnano LC system, coupled to the QExactive Orbitrap MS (all Thermo Fisher Scientific, Vienna, Austria). Peptides were trapped on a C18 2 cm × 100 μm precolumn, and LC separation was performed on a 50 cm × 75 μm Pepmap100 analytical column (both Thermo Fisher Scientific, Austria). Following this, 1 µL of sample was injected. The 85 min HPLC method included a 43 min gradient from 7% to 40% mobile phase B (79.9% acetonitrile, 20% H_2_O, 0.1% formic acid) at a flow rate of 300 nL/min. The resolution on the MS1 level was set to 70,000 (at *m*/*z* = 200), with a scan range from 400 to 1400 *m*/*z*. The top eight abundant peptide ions were chosen for fragmentation at 30% normalized collision energy and resulting fragments were analyzed in the Orbitrap at a resolution of 17,500 (at *m*/*z* = 200).

### 2.12. Proteomics Data Analysis

Raw data were subjected to the freely available software MaxQuant (version 1.6.0.1), utilizing the Andromeda search engine, followed by statistical evaluation with the Perseus software (version 1.6.0.2) [27,28,29,30,31]. For the MaxQuant search, a minimum of two peptide identifications, at least one of them being a unique peptide, was required for valid protein identification. Digestion mode was set to “Specific”, choosing Trypsin/P. The peptide mass tolerance was set to 50 ppm for the first search and to 25 ppm for the main search. The false discovery rate (FDR) was set to 0.01, both on peptide and protein levels, based on the q-value. The database applied for the search was the human Uniprot database (version 03/2018, reviewed entries only), with 20,316 protein entries. Further settings for the search included carbamidomethylation as fixed modification and oxidation of methionine and acetylation of the protein *C* terminus as variable modifications. (Principal Component Analysis, PCA) is provided in Supplementary Figure S1.

### 2.13. UHPLC-MS/MS for Eicosanoid Measurements

Analytes were separated using a Thermo Scientific Vanquish (UHPLC) system and a Kinetex^®^ C18—column (2.6 μm C18 100 Å, LC Column 150 × 2.1 mm; Phenomenex^®^). A 20 min gradient flow method was applied, starting at 35% B and steadily increasing to 90% B (1–10min), going up to 99% B in 0.25 min. Flow rate was kept at 200 µL/min; 20µL injection volume and column oven temperature was set to 40 °C. Eluent A contains H_2_O + 0.2% FA and eluent B ACN:MeOH (90:10) + 0.2% FA

A mass spectrometric analysis was performed, with a Q Exactive HF Quadrupole-Orbitrap mass spectrometer (Thermo Fisher Scientific, Austria), equipped with a HESI source (heated electrospray ionization) for negative ionization. Mass spectra were recorded, operating from 250 to 700 *m*/*z* at a resolution of 60,000 @ 200 *m*/*z* on MS1 level. The two most abundant precursor ions were selected for fragmentation (HCD 24 normalized collision energy), preferentially molecules from an inclusion list, which contained 32 *m*/*z* values specific for eicosanoids. MS2 was operated at a resolution of 15,000 at 200 *m*/*z*. For negative ionization, a spray voltage of 2.2 kV and a capillary temperature of 253 °C were applied, with the sheath gas set to 46 and the auxiliary gas to 10 arbitrary units.

Raw files generated by the Q-ExactiveTM OrbitrapTM were analyzed manually, using Thermo Xcalibur 4.1.31.9 (Qual browser), referring to lipid standards for all described lipids purchased from Cayman (Cayman Europe, Tallinn, Estonia). For peak integration, the software TraceFinderTM (version 4.1—Thermo Scientific, Austria) was used.

### 2.14. In Silico Molecular Modeling

The molecular modeling approach relied on an integrated use of docking, pharmacophore modeling and molecular dynamic (MD) simulations.

Both the 3D structures of AOH and cholesterol were downloaded from the PubChem database (https://pubchem.ncbi.nlm.nih.gov/). Conversely, the caveolin scaffolding domain (CSD) region still lacks a through 3D characterization. Therefore, the portion 86-KASFTTFTVTKYWFYRL-102, which is thought to include the CRAC motif (cholesterol recognition-interaction amino acid consensus), was modeled in silico using the Build Protein module of the software Sybyl v. 8.1 (www.certara.com). Albeit, a consensus on the secondary structure of this region is still missing, the alpha-helix conformation was chosen in agreement with: (i) previous studies addressing the modeling of CRAC domains [32,33]; (ii) evidences pointing to the from-β sheet-to-α helix shift getting close to the central residues of the CRAC motif [17]; (iii) muclear magnetic resonance data highlighting the predominant alpha-helical structure of CRAC [34]; (iv) the sheet-helix transitions observed in CRAC motifs, adjacent to trans-membrane helices [35].

Pharmacophoric analysis. The modeling of pharmacophoric space was done using the GRID module algorithm, implemented in the software Flap (Fingerprint for Ligand And Protein; https://www.moldiscovery.com) [36]. This step aimed at describing, in terms of polar and hydrophobic repartition, the surrounding space available for arranging ligands.

Docking study. The docking study aimed at assessing the mode of binding of at the CRAC domain, in comparison to that of cholesterol, taken as reference. The GOLD software [37] was used to perform all the docking simulations. The software setting parameters and docking procedures reported by Dellafiora and co-workers were used [38].

MD simulations. The best scored pose of AOH underwent MD simulations over 50 nsec to assess the capability of AOH to geometrically persist at the CRAC domain during the considered timeframe. In particular, MD simulations were performed using GROMACS (version 5.1.4) [39], with CHARMM27 all-atom force field parameters support [40], in agreement with a previous study [41] Briefly, protein-peptides complexes were solvated with SPCE waters (electrochemical screen printed carbon electrodes) in a cubic periodic boundary condition, and counter ions (Na^+^ and Cl^−^) were added to neutralize the system. Prior to MD simulation, the systems were energetically minimized to avoid steric clashes and to correct improper geometries using the steepest descent algorithm with a maximum of 5000 steps. Afterwards, all the systems underwent isothermal (300 K, coupling time 2 psec) and isobaric (1 bar, coupling time 2 psec) 100 psec simulations, before running 50 nsec simulations (300 K with a coupling time of 0.1 psec and 1 bar with a coupling time of 2.0 psec).

## 3. Results

### 3.1. Crosstalk between AOH and LPS, with Regard to the Secretome of THP-1 Macrophages

It was previously demonstrated that AOH may suppress pro-inflammatory induction triggered byLPS [3,6]. We took advantage of untargeted proteome profiling, and investigated the effects of LPS and/or AOH on the secretome profile in THP-1 macrophages. At a sub-cytotoxic concentration (1 µM, Supplementary Figure S2), AOH alone did not elicit any detectable response in the secretome profile of the macrophages (Figure 1B, 5 h incubation). LPS treatment resulted in the significant regulation of 19 proteins in THP-1 macrophages (Figure 1B–D). However, a combination of the two treatments (LPS and AOH) reduced the number of proteins regulated by LPS to 6 (Figure 1B,D). These 6 proteins were found consistently regulated in presence or absence of AOH, and all of them are pro-inflammatory mediators (Figure 1D). This result suggested that the macrophages could retain the capability to release pro-inflammatory mediators in response to LPS, even in the presence of AOH and opened the possibility that the crosstalk between the two could occur downstream from this point.

### 3.2. Effect of AOH on the Membrane of THP-1 Macrophages

**Figure 2:**
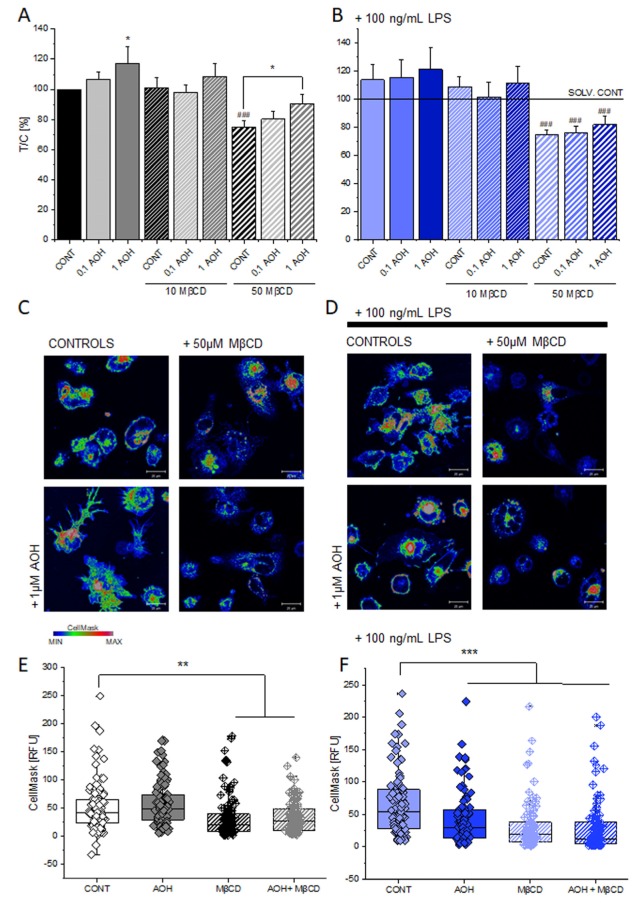
Effect of AOH (0.1–1 μM) and MβCD (10–50 μM) on the membrane fluidity of THP-1 macrophages at indicated conditions (**A**) and in additional presence of 100 ng/mL LPS (**B**). Data are mean ± SEM of eight independent experiments performed in triplicate. ### (*p* < 0.001) identifies significant decrease, according to the ANOVA test with Fisher test in comparison to controls. The appearance of the membrane of THP-1 macrophages (**C**,**D**) after 1 h incubation with the compounds of interest and staining with CellMask for cell membrane. Scale bar: 20 µm. Quantification of fluorescence intensity of the cell membrane staining (RFU, Relative Fluorescence Units; 1 μM AOH, 50 μM MβCD or 1 μM AOH + 50 μM MβCD; (**E**,**F**)). Data are obtained from the quantification of n ≥ 80 cells from four independent experiments and ** and *** indicate significant difference in comparison to controls with *p* < 0.01 and *p* < 0.001 with Student’s *t*-test.

Considering the response capability of THP-1 macrophages to combined LPS and AOH stimulation, we pursued the hypothesis that AOH could impair signal transduction of the pro-inflammatory cascade. As the response to LPS is mediated by trans-membrane receptors [16,42] and membrane oxidative status is considered crucial for the propagation of the inflammatory cascade [21], the effects of the toxin on the membrane biophysical properties and morphology were investigated. Membrane fluidity was found to be increased in a concentration dependent fashion in the presence of the mycotoxin and this effect was counteracted by the presence of the cholesterol-complexing agent methyl-beta cyclodextrin [43] (MβCD, Figure 2A). The presence of LPS caused a general increase of membrane fluidity and possibly reduced the visibility of the competition between AOH and MβCD (Figure 2B). Since measurement of the membrane fluidity is technically limited to a reduced incubation time (10 min) [23], the morphological response of the cell membrane was also monitored (1 h incubation, Figure 2C,D). Appearance of the cell membrane (evaluated as intensity of the CellMask staining) was altered by incubation with MβCD, but no effect could be attributed to the mycotoxin AOH (Figure 2C–E). In the presence of LPS, macrophages increased their spreading area, as previously also described upon activation in other models [44]. The co-incubation with AOH and/or MβCD reduced this response and possibly also influenced the lipid reorganization, which is typically associated to it. This phenomenon could be followed also as a decrease of the signal of the cell membrane fluorescence tracking (Figure 2D–F).

### 3.3. Effect of AOH on Mitochondrial Superoxide

**Figure 3:**
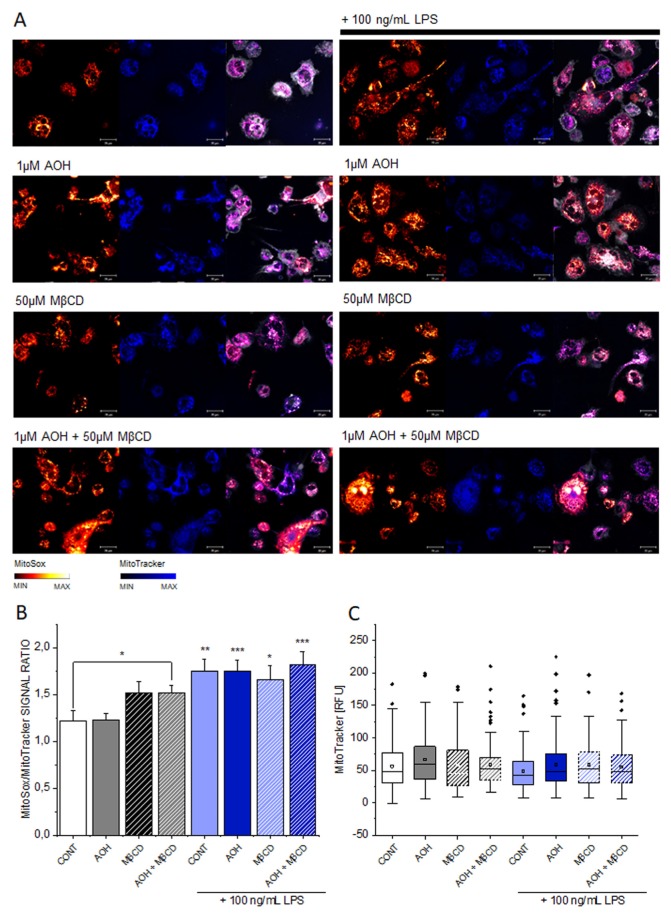
Mitochondrial superoxide response in THP-1 macrophages. Signal intensities of MitoSox range from orange to white, of MitoTracker from black to blue (see color bars) and cell membraneintensities are depicted in white (**A**). MitoSox/MitoTracker signal ratio measured after 1 h incubation (**B**). Intensity of the mitochondrial signal as reference (**C**). Incubation refer to 1 μM AOH, 50 μM MβCD or combination of the two in presence or absence of LPS 100 ng/mL. Scale bar, 20 µm. Data are mean ± SEM n ≥ 80 cells from 4 independent experiments. * (*p* < 0.05), ** (*p* < 0.01), *** (*p* < 0.001) significant difference according to Student’s *t*-test in comparison to controls.

In order to verify if the effects of AOH on membrane fluidity could be associated to oxidative stress and reactive oxygen species (ROS) increase [23], the potential of AOH to induce superoxide ion production was measured via mitochondrial ROS formation. It is well known that mitochondrial ROS may impact on lipid peroxidation and consequently on membrane biophysical properties [45], as well as play a central role in inflammatory cascades [46,47]. In all conditions, the treatment with LPS was associated with an increase of mitochondrial superoxide, while no effect could be attributable to AOH alone (Figure 3). Relevance of the quantification, expressed as MitoSox/MitoTracker signal intensity ratio (Figure 3B), was confirmed by the constant signal of MitoTracker (blue, Figure 3C). Hence, differently to LPS, the effects on the lipid membrane triggered by AOH seemed to be independent from pathways regulating the mitochondrial superoxide production.

### 3.4. Crosstalk between AOH and MβCD on the Secretome of THP-1 Macrophages

**Figure 4:**
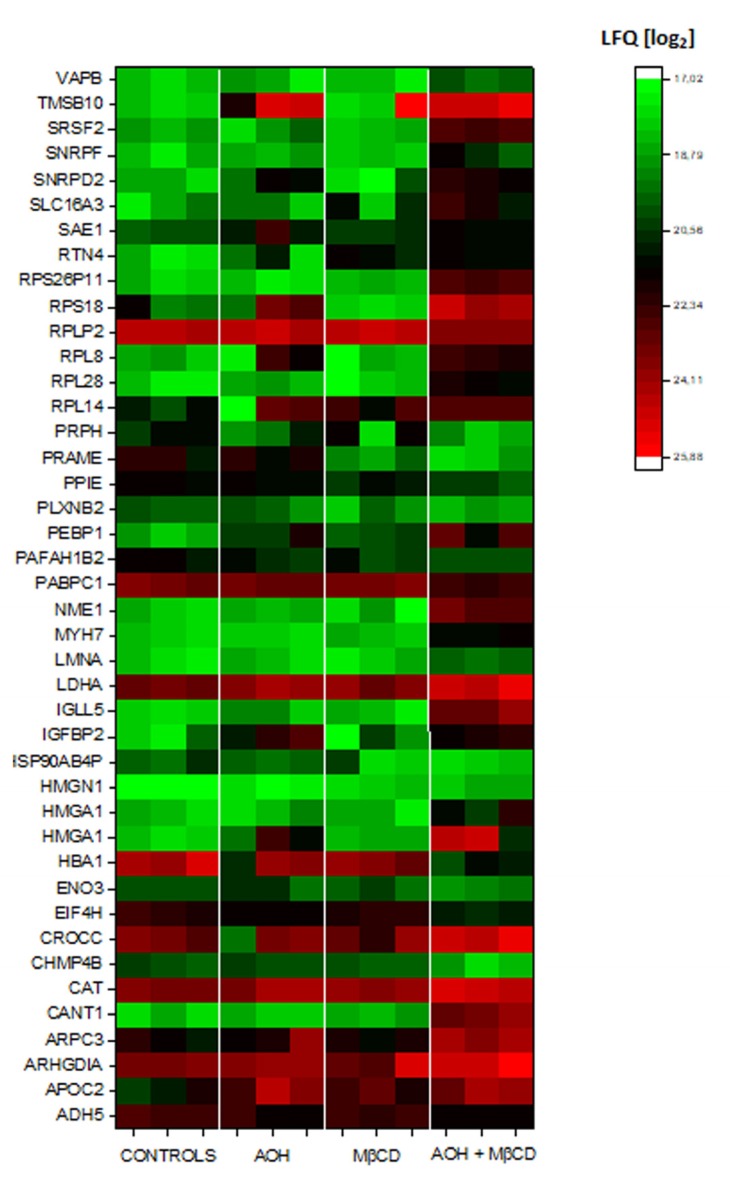
Secretome analysis of THP-1 macrophages incubated with AOH (1 μM) and/or MβCD (50 μM) for 5 h. Heat map depict the 3 biological replicates performed for each treatment group and includes all the proteins significantly regulated by the combination of 1 μM AOH + 50 μM MβCD. Single treatment (1 μM AOH or 50 μM MβCD alone) induced no significant regulation in comparison to controls. Green to Red colors depict the variation of the LFQ intensities [log_2_].

In order to deeper investigate the cross-talk between AOH and MβCD, the analysis on the secretome of THP-1 was extended with the respective experimental conditions. As for 1µM AOH, secretome analysis revealed no significantly regulated proteins in macrophages incubated with 50µM MβCD (Figure 4). However, the combination of the two compounds triggered the significant regulation of more than 40 proteins (Figure 4). Among these, we identified proteins important for the metabolism and cellular structural organization like monocarboxylate transporter 4 (SLC16A3), insulin-like growth factor-binding protein 2 (IGFBP2). Moreover, the nucleoside diphosphate kinase A (NME1, involved in the phosphorylation of membrane proteins [48]) was found consistently up-regulated as well as rho GDP-dissociation inhibitor 1 (ARHGDIA). Rho GDP-dissociation inhibitor is essential for the homeostasis of Rho GTPases [49] and plays a central role in the regulation of the expression of COX-2 [50]. Same behavior could be measured for Thymosin beta-10 (TMSB10) and this can be possibly related to the morphological change of the THP-1 cells upon treatment, as previously already reported also in breast cancer cells [51]. Similarly, the release of the cytoskeletal binding protein Rootletin (CROCC) [52] also increased, whereas the charged multivesicular body protein 4b (CHMP4B) was significantly down-regulated. CHMP4B belongs to the endosomal-sorting complex required for transport (ESCRT) that regulates membrane budding and exosomes formation [53] (Figure 4). Overall, many regulatory events seemed to have the membrane and membrane bound proteins as common denominator thus reinforcing the hypothesis that AOH could act at this level in THP-1 macrophages.

### 3.5. Computational Study of Molecular Interactions between AOH and Cholesterol

**Figure 5:**
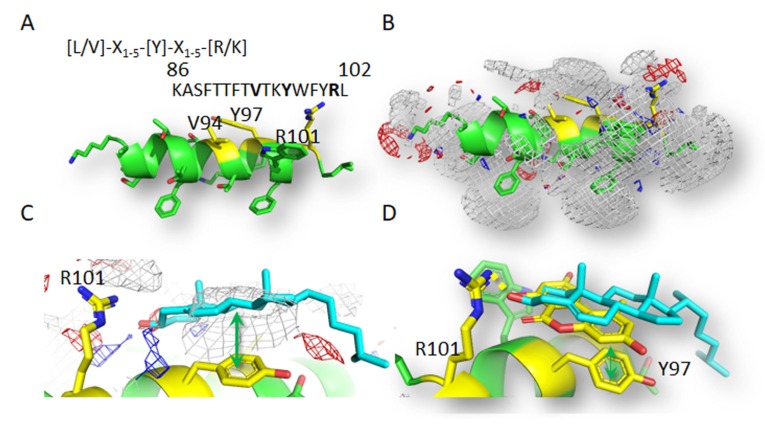
CRAC motif and the modeled CRAC portion of caveolin 1’s CSD domain. (**A**) CSD sequence containing the CRAC motif is shown and the conserved residues are highlighted in bold. (**B**) The protein is represented in sticks and cartoon, while gray, red and blue meshes indicate regions sterically and energetically able to receive hydrophobic, H-donor acceptor and H-bond donor groups, respectively. (**C**) Calculated interaction of cholesterol (represented in cyan sticks) at the CRAC portion of caveolin 1’s CSD domain. The protein is represented in sticks and cartoon, while gray, red and blue meshes indicate regions sterically and energetically able to receive hydrophobic, H-donor acceptor and H-bond donor groups, respectively. The green arrow indicates the formation of hydrophobic stacking, while the yellow dotted line indicates the formation of polar interaction. (**D**) Calculated interaction of AOH (represented in yellow sticks) overlapped to the calculated pose of cholesterol (represented in cyan sticks) at the CRAC portion of caveolin 1’s CSD domain. The green arrow indicates the formation of hydrophobic stacking, while the yellow dotted line indicates the formation of polar interactions.

Since the cross-talk between MβCD and AOH appeared to be centered at membrane level, affecting membrane biophysical properties as well as the secretome of THP-1 macrophages, we pursued the hypothesis that immunomodulatory action of the mycotoxin could be related to a direct effect with membrane structures/receptors. Since the effects of AOH on THP-1 pointed toward a competition with cholesterol, we decided to investigate the likelihood of AOH to interact with typical membrane structures containing cholesterol. Particularly, caveolin 1 allows the binding of cholesterol [54], and homeostasis of the protein is gaining more and more importance in the regulation of inflammatory processes [55,56,57,58], as well as acting as a cell mechanosensor and metabolic regulator and scaffolding domain for intracellular proteins [59,60,61]. In order to clarify the binding potential of AOH, a pharmacophoric analysis of the CRAC portion of the CSD domain of caveolin 1 was performed. This approach showed a prevalence of hydrophobicity in the space deputed to bind cholesterol (Figure 5), in agreement with its marked hydrophobicity. The docking study provided a plausible binding architecture of cholesterol that was found engaging R101 with the hydroxyl group, while the polycyclic hydrophobic core was found embedded into the surrounding hydrophobic space. In more detail, hydrophobic stacking between Y97 and the C ring was found (Figure 5), in agreement with previous studies on cholesterol-CRAC motif interaction [32]. AOH showed a binding pose retracing the one of cholesterol (Figure 5), along with recording a very close computational score (AOH = 39.693, cholesterol = 39.697), that might point to its comparable capability to satisfy the space available for receiving ligands, according to previous studies [62]. In particular, the hydroxyl group in position #7 was found engaged in a polar interaction with R101, while aromatic rings were found engaged in hydrophobic stacking with Y97. The capability of the AOH-CRAC motif interaction to persist over time was assessed by means of MD simulations. The interaction of AOH was found to not interfere with the overall peptide geometry, as testified by the quite stable RMSD values (root mean square deviation values), recorded along the whole simulation (Supplementary Figure S3A). This result may point to the capability of AOH to geometrically stabilize the CRAC portion of the CSD domain of caveolin-1. In addition, AOH showed a discrete reducing trend in the RMSD values along the simulation (Supplementary Figure S3B), suggesting stable interaction with the CRAC motif. Furthermore, the cluster analysis of AOH trajectory identified six geometrical clusters (RMSD cutoff 0.75 Å; Supplementary Figure S4), and five of them encompassed interactions with at least one of the conserved residues of the CRAC motif. This result supports the interpretation that multiple interacting modes might exist in the case of isolated peptides bearing CRAC motifs. Notably, interactions with W98 were found recurrent, implying its importance in the AOH-CRAC interaction, even though it is not included in the set of conserved residues. It is worth noticing that the polar engagement of R101 was not found in any of the calculated clusters, reflecting the minor recurrence of poses prone to form such a contact along the all simulation. Given the marked recurrence of non-polar contacts in all the clusters, these results may suggest that hydrophobic interactions have a major role in driving the interaction with the CRAC motif.

### 3.6. Effect of AOH and LPS on Caveolin-1 and TLR4 in THP-1 Macrophages

**Figure 6:**
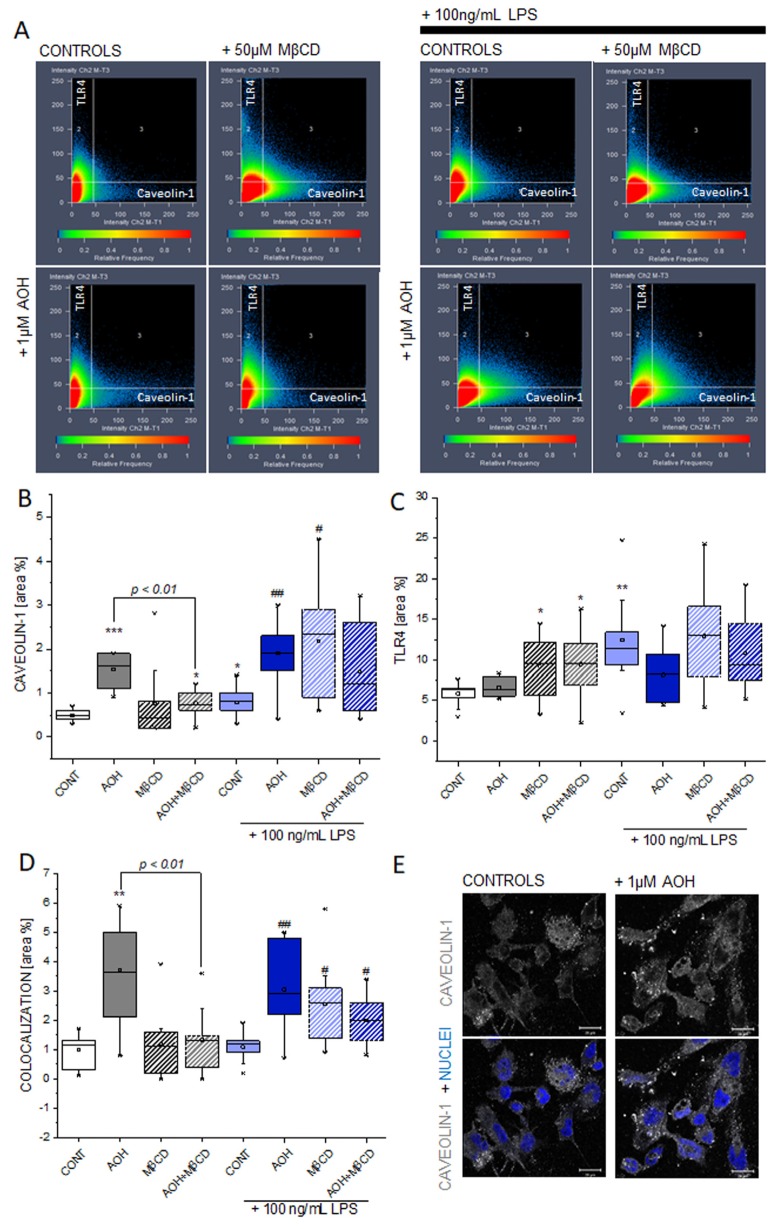
Influence of AOH on the immunolocalization of caveolin-1 and TLR4. Signal intensity and co-localization of the fluorescent signals (**A**). Area of caveolin-1 signal (**B**), TLR4 (**C**) and co-localization (**D**), after 1 h incubation with 1 μM AOH, 50 μM MβCD, or both in presence or absence of 100 ng/mL LPS. Appearance of the immunolocalization of caveolin-1 in THP-1 macrophages in control conditions and after 1 h incubation with 1 μM AOH (**E**). Scale bar, 20 µm. Data are mean of n ≥ 8–9 optical fields obtained from three independent experiments, * (*p* < 0.05), ** (*p* < 0.01), *** (*p* < 0.001) and # (*p* < 0.05), ## (*p* < 0.01) indicate significant differences in comparison to controls (CONT) and CONT + LPS, respectively (Mann–Whitney test).

Since AOH seems to have the potential to substitute cholesterol in membrane proteins like caveolin-1, we decided to investigate the correlation with the transmembrane receptor TLR4. TLR4 activation is central for the LPS-induced pro-inflammatory cascade [63] and immunofluorescence confocal microscopy followed by an image analysis allowed one to localize and quantify the receptors of interests. TLR4 signal area increased significantly after incubation with LPS, as well as by incubation with MβCD and MβCD/AOH (Figure 6A,C). The signal of caveolin-1 increased after incubation with AOH (Figure 6A,B,E) and this effect was significantly reduced by the co-incubation with MβCD (Figure 6A,B). The signal of caveolin-1 increased upon incubation with AOH also in presence of LPS (Figure 6A,B). The co-localization analysis showed an increase the area TLR4/caveolin-1, in association with the AOH incubation. This effect was decreased by the addition of MβCD (Figure 6D). In LPS-stimulated cells, the effect was visible, albeit to a lesser extent, also in the presence of MβCD and MβCD/AOH (Figure 6A,D). All in all, these data point toward the capability of the mycotoxin AOH to modify the cellular localization of caveolin-1, as well as its crosstalk with TLR4.

### 3.7. Effect of AOH on the PUFA and Oxylipin Profile of THP-1 Macrophages

**Figure 7:**
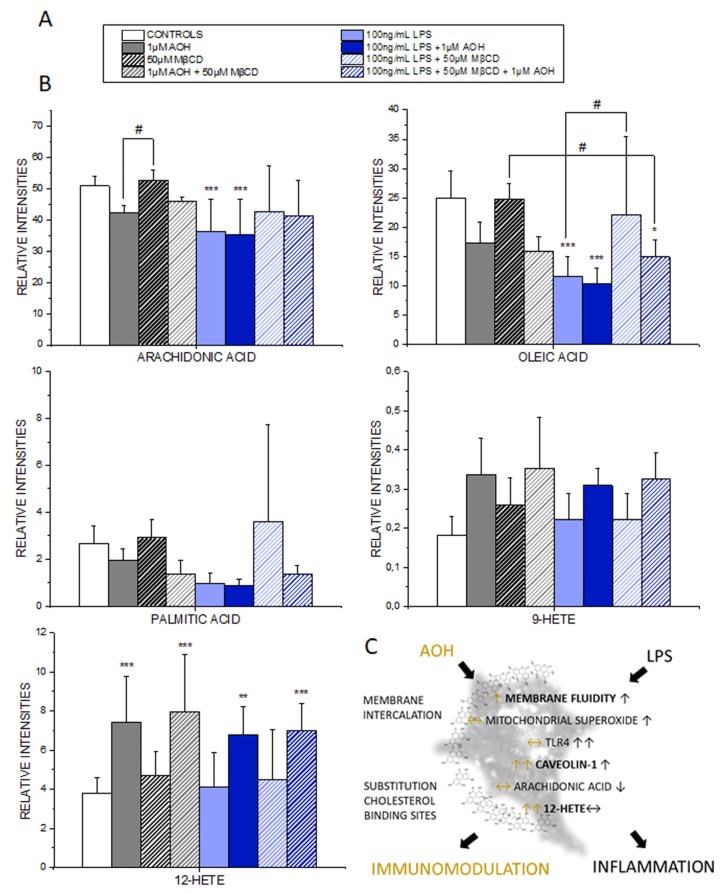
Influence on arachidonic, oleic and pamititc acid and on 9-and 12-HETE in the secretome of THP-1 macrophages (3 h incubation). (**A**) Figure color legend. (**B**) Data are depicting results obtained from 3 independent cell preparations. * (*p* < 0.05), ** (*p* < 0.01), *** (*p* < 0.001) indicates significant differences in comparison to controls and # (*p* < 0.05) indicates significant differences among the treatments (ANOVA for multiple comparisons). (**C**) Schematic representation of the mechanism of immunomodulatory action of AOH.

Since more and more evidence point toward an effect of AOH centered at membrane level, we investigated the effects of the mycotoxin on other key regulators of the immune response, whose activity is centered on the lipid membrane, namely hydroxyeicosatetraenoic acids (HETEs) and PUFAs. An untargeted approach allowed one to obtain an overview on the effects on lipid mediators, as well as on their precursors. LPS significantly decreased the concentration of arachidonic acid and oleic acid in the medium, indicating the increased enzymatic processing of these precursor molecules (Figure 7A,B). Intriguingly, this effect was also maintained also by the co-incubation LPS + AOH, sustaining the hypothesis that when the two stimuli are applied together, the macrophages should maintain the capability to produce pro-inflammatory mediators. A tendency toward decrease was observed in parallel in all the incubation conditions that included AOH. Moreover, the incubation with MβCD alone did not elicit any effect, thus confirming on one side the data already observed with the secretome at protein level, and implying on the other that AOH can have a specific effect on the lipid components. As for the HETEs, AOH selectively induced the formation of 12-HETE, thus implying a possible increase of the 12-HETE-associated signals when THP-1 macrophages are incubated with the toxin. A similar trend, albeit not significant, was observed for 9-HETE (Figure 7B). Of note, 12-HETE can sustain transcription of proinflammatory mediators [64], thus adding another level to the interpretation of the biological effects of AOH. In line, immunostaining of the cytokine MIF revealed an increase when macrophages were incubated with AOH, thus suggesting that the mycotoxin alone could also potentially trigger some pro-inflammatory responses (Supplementary Figures S5–S7).

## 4. Discussion

The description of mechanisms sustaining the immunomodulatory potential of environmental contaminants is of crucial importance; especially when exposure occurs in a domestic context, where the possibility to contain/decrease contact can be very limited, as well as risk perception. We are progressively gaining a better understanding of the toxicological potential of the exposure to molds and the subsequent relation with pathologies of the respiratory tract [1,2,9]. However, causal relationships and respective treatment opportunities require the elucidation of the molecular mechanisms sustaining these effects. Even though AOH was typically considered as immunosuppressive of the LPS-induced pro-inflammatory cascade [3,6], an untargeted secretome analysis of the supernatant of macrophages incubated with LPS or LPS + AOH showed a very similar profile (Figure 1). Indeed, we obtained a comparable induction of interleukin 1 beta, the TNF receptor-associated factor 1 and tumor necrosis factor alpha-induced protein 2, as well as from C-C motif chemokine 20. In this light, when co-incubated with LPS, AOH did not seem to alter cell capability to produce pro-inflammatory signals, or at least not within a timeframe of 5 h. This observation guided us toward the idea that AOH could impair signal transduction in macrophages, rather than signal generation. Pursuing this hypothesis, we compared the effect of the toxin at membrane level with that of LPS, and were able to observe, in both cases, the alteration of membrane biophysical properties (fluidity, Figure 2). However, further experiments suggested that the same result could be mediated by the activation of differential pathways. In the case of LPS, increase of membrane fluidity was parallel to the increase of the superoxide formation at mitochondrial level (Figure 3), being ROS formation a typical response of immune cells upon activation [47]. Intriguingly, AOH reduced also the morphological adaptation of macrophages in response to LPS (Figure 2), thus retracing the behavior of the polyphenolic flavonoid silymarin that, exactly like AOH [6] can block the activation of the transcription factor NF-ĸB as well as the LPS-induced morphological adaptation of RAW264.7 cells [65]. In our experimental conditions, we also observed that AOH was capable to compete with the cholesterol complexing agent MβCD in the modulation of membrane biophysical properties, without the involvement of the mitochondrial activation (Figure 3). This crosstalk suggested that cholesterol containing structures like caveolae and lipid rafts [66] could be important players in the mechanism of action of AOH. Moreover, these results supported previous work describing an increased membrane fluidity triggered by AOH (30 μM, 24 h incubation) and a parallel alteration of the distribution of the GM1 plasma membrane raft ganglioside in RAW264.7 macrophages [5]. To sustain a potential connection with the immunomodulatory activity of AOH, it was previously demonstrated that cholesterol depletion with MβCD and caveolin-11 silencing abolish IL1β-mediated MAPK-p38 signal transduction, and that tumor necrosis factor receptor-associated factor 6 (TRAF6) was essential for the activation of this pathway [67] A protein secretome analysis confirmed the potential crosstalk between AOH and MβCD. If none of the two compounds alone elicited the variation of secreted protein profiles in comparison to controls, the combination of the two significantly regulated more than 40 proteins. Depletion of membrane cholesterol via MβCD is known to modify the caveolar-proteome [68] making it plausible that combination of the two treatments (AOH + MβCD) could potentiate the biological response at this level. Among the significantly regulated proteins, several presented a link with membrane domains, like, for instance, the monocarboxylate transporter (MCT4; SLC16A3), whose expression was found to relate to that of caveolin-1 in tumor stroma [69]. Similarly, the Rho GDP-dissociation inhibitor 1 (ARHGDIA) was found to be up-regulated in the secretome by the co-incubation of AOH and MβCD. The Rho GDP-dissociation inhibitor 1 was described to localize with TNRF1 at the membrane level and to be involved in the TNF-α –Rho activation in a cholesterol/caveolin 1 dependent manner [70].

In line, alteration of the membrane homeostasis would impair the signal transduction in presence of pro-inflammatory stimuli. It was previously demonstrated that cholesterol loading via MβCD increases the stability of caveolin-1 and its localization in the cell membrane [54]. In line with the hypothesis that AOH could structurally interact with the CRAC binding domain of caveolin 1 as suggested by the modelling experiments (Figure 5), incubation of THP-1 macrophages with AOH significantly increased the staining intensity of caveolin-1. The caveolin-1 response was accompanied by an increase in the co-localization with TLR4 (Figure 6A,D). These data are in accordance with previous studies describing the recruitment of TLR4 dependent from caveolin-1/FABP7 in astrocytes [71], thus providing a mechanistic insight into a possible immunomodulatory activity of the toxin also in the presence of pro-inflammatory cytokines and chemokines (as suggested in Figure 1). In accordance with the interpretation that AOH could compete with cholesterol at membrane level, a lipidomic profiling of the secretome was also performed. It was previously demonstrated that membrane proteins like caveolin-1 are crucial in the homeostasis of long chain fatty acids [72], and similarly, in our model, the processing of arachidonic acid, oleic acid and palmitic acid appeared modulated by the presence of AOH (Figure 7). Similarly, macrophages from caveolin-1 null mice were reported to exhibit elevated arachidonic acid uptake [73], and even though a postulated stabilization of caveolin-1 from AOH at membrane level could be associated with a gain or a loss of function, the consistency of the effect/trend on the precursors is coherent with the interpretation that AOH could act at this level. In agreement with the interpretation that AOH could stabilize the presence of caveolin-1, PUFAs show lower concentrations in presence of AOH, in comparison to incubation conditions without mycotoxin (Figure 7) [72,74].

According to the molecular modelling experiments, AOH has the capability to bind to the CRAC domain of caveolin, with a scoring similar to that of cholesterol itself (Figure 5). Although topological and geometrical changes of the portion calculated cannot be ruled out in the real protein context, the results presented here were all in agreement with the capability of AOH to interact favorably with the CRAC portion of caveolin 1’s CSD domain. On this basis, the interaction with the CSD at the CRAC locus was proposed, to mechanistically explain the possible AOH-caveolin 1 interaction observed experimentally. A structural similarity between AOH and related compounds with cholesterol is at the basis of the capability of the mycotoxin to act as mycoestrogen and bind to the estrogen receptors [75]. Similarly, the stabilization of caveolin-1 at membrane level through AOH could possibly modulate inflammatory cascade via multiple mechanisms, such as, for instance: (i) altered turnover of the TLR4, (ii) altered association with TRAF1 and IL1B (iii) altered uptake of eicosanoid precursors. Considering the central role of the cell membrane in mediating the transduction of the pro-inflammatory signal, this would provide a mechanistic model to explain the immunomodulatory action of AOH when incubated with LPS. At the same time, we would not exclude that in some contexts the toxin could also trigger pro-inflammatory signals, thus also confirming respective literature describing the pro-inflammatory potential of AOH [12]. Structural similarities between AOH and cholesterol include the possibility of direct interaction/intercalation of the toxin in the plasma membrane, also outside caveolae. On a similar principle, structural analogy is at the bases of the capability of endocannabinoids to embed into membrane and increase their fluidity, as well as to consequently regulate lipoxygenase binding [76]. Similarly, membrane fluidity was reported to be an important modulator of the activity of the 5-lipoxygenase [20]. In line, also non-steroidal anti-inflammatory drugs—NSAIDs are able to modulate membrane fluidity, thus supporting the molecular mechanism of the drugs on the cyclooxygenase [77,78]. On the basis of the capability of AOH to increase membrane fluidity (Figure 2), this could be sufficient to support an effect on the inflammatory cascade. Accordingly, the potential effect of AOH on the eicosanoids production could be postulated as dependent or independent from the effect on the caveolae system. All incubation conditions, including AOH, significantly increased the 12-HETE, and a similar tendency was observed regarding 9-HETE. In agreement we formulated the idea that the similarity between AOH and cholesterol could be a key to understand the immunomodulatory potential of the mycotoxin, and its mechanism of action at membrane level (Figure 7C). Cholesterol plays also an essential role in the activation of the NLRP3 inflammasome activation [79], as well as in transducing lipid peroxidation signals in macrophages [23]. In agreement, the effects of AOH on the immunodetection of caveolin-1 (Figure 6), and of the cytokine MIF (Supplementary Figures S5 and S7), were reduced by the cholesterol deprivation with MβCD, exactly like the membrane fluidity response (Figure 2).

## 5. Conclusions

In conclusion, we described the effect of the mycotoxin AOH on several aspects of THP-1 macrophage functions. As for the interaction with the estrogen receptor and the respective effect as xenoestrogen [75,80], also for the immunomodulatory potential of AOH, the structural similarity of the toxin with endogenous molecules like cholesterol may provide the key for understanding its complex biological functions. This could be obtained either through direct interaction with the membrane, or through interaction with cholesterol containing proteins like caveolin-1 (Figure 7C).

## Supplementary Materials

The following are available online at https://www.mdpi.com/2073-4409/9/4/847/s1, Figure S1: Principal Component Analysis (PCA) of the secretome analysis Figure S2: Evaluation of the cytotoxic potential of the incubation with AOH; Figure S3: Geometrical changes of the CRAC portion of caveolin 1’s CSD domain and AOH; Figure S4: sters identified by the cluster analysis; Figure S5: Representative images of the immunolocalization of Caveolin-1, macrophage migration inhibitory factor, toll like receptor 4 in THP-1 macrophages; Figure S6: Representative images of the immunolocalization of Caveolin-1, macrophage migration inhibitory factor, toll like receptor 4 in THP-1 macrophages; Figure S7: Effect of 1 h incubation with 1 μM AOH and/or 50 μM MβCD on the pro-inflammatory cytokine MIF.
