# Supplementary material for: Structural Similarity with Cholesterol Reveals Crucial Insights into Mechanisms Sustaining the Immunomodulatory Activity of the Mycotoxin Alternariol
Source: Cells. 2020 Mar 31;9(4):847. doi: 10.3390/cells9040847 (PMC7226804; doi:10.3390/cells9040847)

Structural similarity with cholesterol reveals crucial insights into mechanisms sustaining the immunomodulatory activity of the mycotoxin alternariol

Giorgia Del Favero [a,b], Raphaela M. Mayer [a], Luca Dellafiora [c], Lukas Janker [d], Laura Niederstaetter [d], Chiara Dall'Asta [c], Christopher Gerner [b,d,e]*, Doris Marko [a,b]*

SUPPLEMENTARY MATERIALS

Figure S1

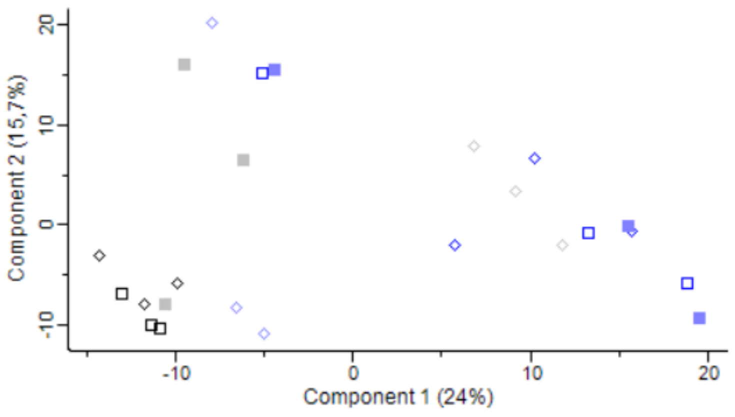

Figure S1. Principal Component Analysis (PCA) of the secretome analysis. White squares black frame CONTROLS. Grey squares 1µM AOH, White diamonds black frame 50µM MβCD. White diamonds grey frame 50µM MβCD + 1µM AOH. White squares blue frame LPS 100ng/mL, Violet squares 1µM AOH + LPS 100ng/mL. White diamonds blue frame 50µM MβCD + LPS 100ng/mL and white diamonds violet frame 50µM MβCD + LPS 100ng/mL + 1µM AOH.

Figure S2

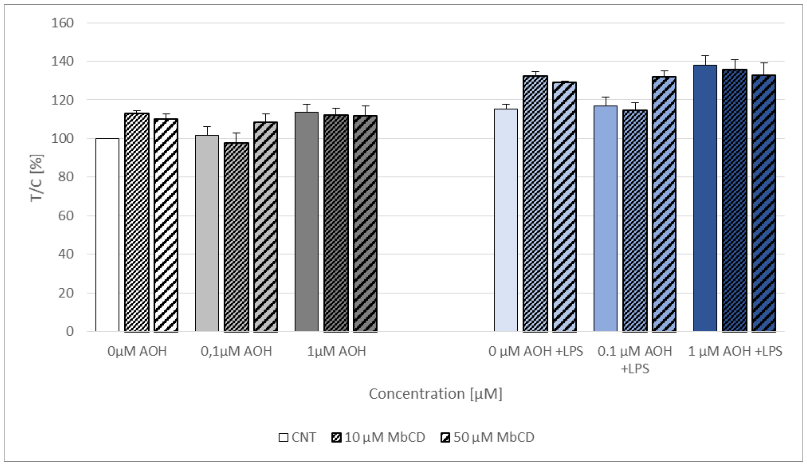

Figure S2. Evaluation of the cytotoxic potential of the incubation with AOH (0.1-1µM, grey bars), MβCD (10-50µM, striped bars) and LPS (100ng/mL, blue bars). Cell viability was carried out in the same experimental conditions as for the secretome analysis in order to exclude artefacts related to cell death. Cells were incubated with WST-1 Reagent (Roche) diluted 1:10 in serum-free RPMI1640 for two hours. Absorbance was measured at 440 nm using a reference wavelength of 650 nm with a Cytation™ 3 Cell Imaging Multi-Mode Reader (BioTek Instruments, Inc.,

[a] Department of Food Chemistry and Toxicology, Faculty of Chemistry, University of Vienna Währinger Straße 38-40, 1090, Vienna, Austria
* Correspondence to : giorgia.del.favero@univie.ac.at and to christopher.gerner@univie.ac.at
[b] Core Facility Multimodal Imaging Faculty of Chemistry, Uniersity of Vienna Währinger Straße 38-40, 1090, Vienna, Austria
[c] Department of Food and Drugs, University of Parma
[d] Department of Analytical Chemistry, Faculty of Chemistry, University of Vienna, Währinger Straße 38, 1090, Vienna, Austria
[e] Joint Metabolome Facility, Faculty of Chemistry, University of Vienna. Währinger Straße 38-40, 1090, Vienna, Austria

Winooski, Vermont, USA). Data are expressed of Treatment/Controls [%] and are the mean of n=5 independent experiments measured in technical triplicates.

Figure S3

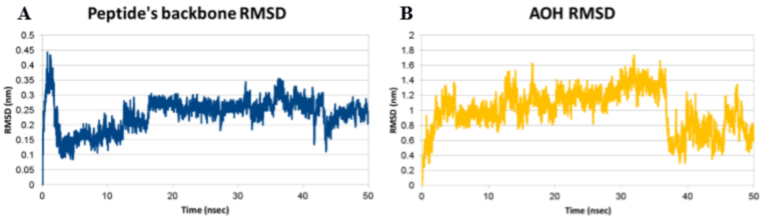

Figure S3. Geometrical changes of the CRAC portion of caveolin 1's CSD domain (A) and AOH (B).

Figure S4

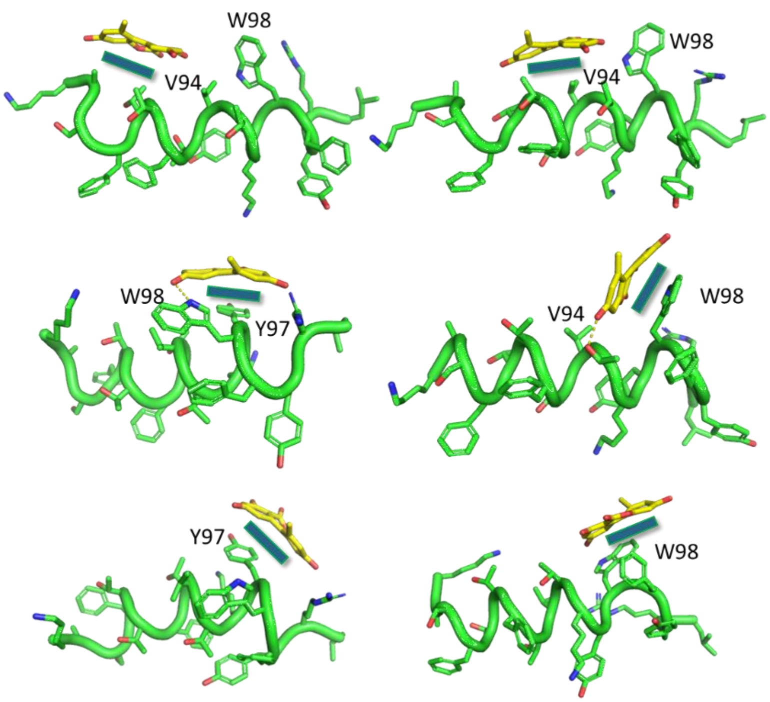

Figure S4. Clusters identified by the cluster analysis (RMSD cutoff 0.75 Å). The peptide is represented in ribbon and sticks (green) while AOH is represented in yellow sticks. Yellow dotted lines indicate polar contacts while green bars indicate the formation of hydrophobic interactions.

Figure S5

Figure S5: Representative images of the immunolocalization of Caveolin-1 (grey), macrophage migration inhibitory factor (MIF, red), toll like receptor 4 (TLR4, green) in THP-1 macrophages. Cell nuclei are counterstained in blue with DAPI. Quantification of the signals of caveolin-1 and TLR4 are reported in figure 6. Scale bars stand for 10 μm.

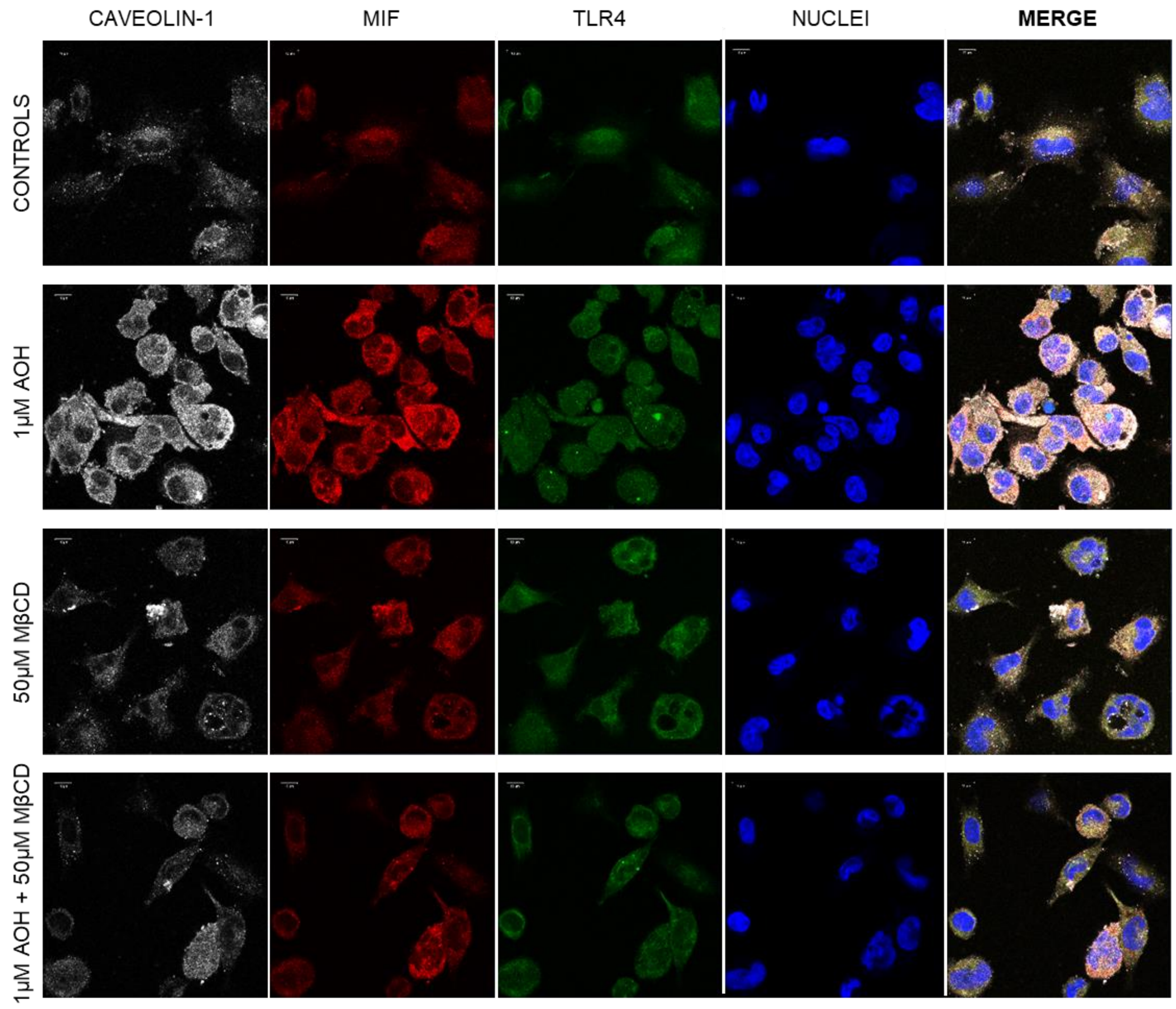

Figure S6

Figure S6: Representative images of the immunolocalization of Caveolin-1 (grey), macrophage migration inhibitory factor (MIF, red), toll like receptor 4 (TLR4, green) in THP-1 macrophages. Cell nuclei are counterstained in blue with DAPI. Quantification of the signals of caveolin-1 and TLR4 are reported in figure 6. Scale bars stand for 10 μm.

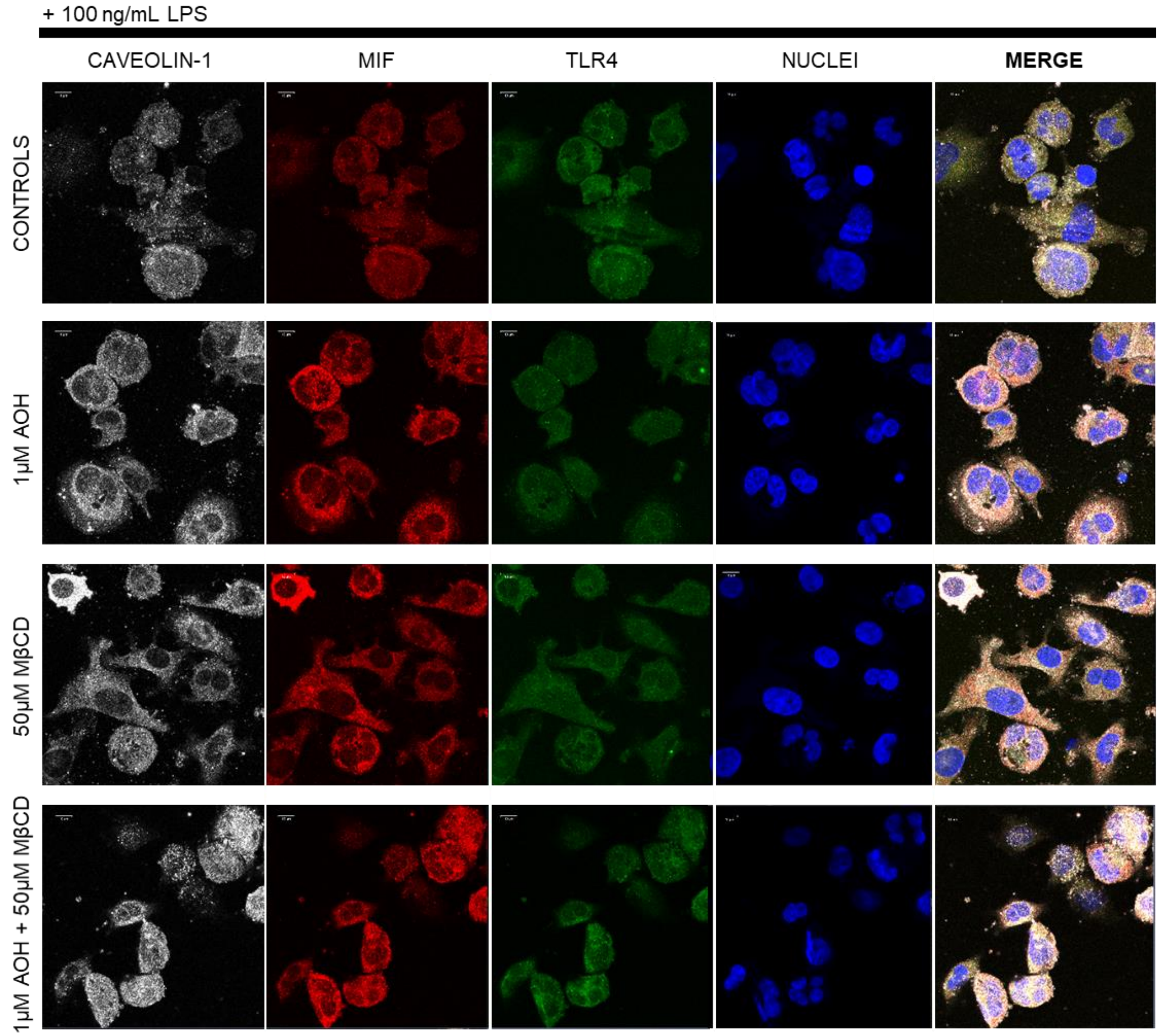

Figure S7

Figure S7: A. Effect of 1h incubation with 1 µM AOH and/or 50µM MβCD on the pro-inflammatory cytokine MIF. B. Effect of 1h incubation with 1 µM AOH and/or 50µM MβCD on the pro-inflammatory cytokine MIF in presence of 100ng/mL LPS. n=8-9 optical fields, from 3 independent experiments * indicates significant differences in comparison to controls (CONT) Mann-Whitney test).

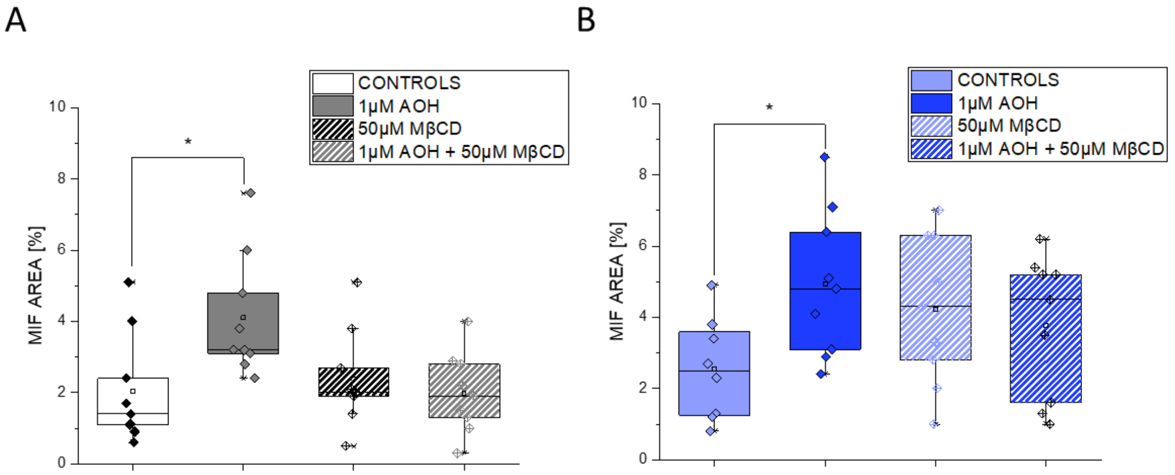

Supplement: Supplementary file 1 [file cells-09-00847-s001.pdf]
